# Characterization of a novel glucocorticoid-resistant human B-cell acute lymphoblastic leukemia cell line, with AMPK, mTOR and fatty acid synthesis pathway inhibition

**DOI:** 10.1186/s12935-021-02335-7

**Published:** 2021-11-25

**Authors:** Yuanyuan Li, Chuan Zuo, Ling Gu

**Affiliations:** 1 Laboratory of Hematology/Oncology, Department of Pediatrics, Key Laboratory of Birth Defects and Related Diseases of Women and Children (Sichuan University), Ministry of Education, West China Second University Hospital, Sichuan University, No.20, Section 3, Renmin South Road, Chengdu, 610041 People’s Republic of China; 2 Joint Laboratory of West China Second University Hospital, Sichuan University and School of Life Science, Fudan University for Pulmonary Development and Disease, Chengdu, 610041 China; 3 NHC Key Laboratory of Chronobiology, Sichuan University, Chengdu, 610041 China; 4 Academic Affairs Department, West China Hospital/West China School of Medicine, Sichuan University, Chengdu, China

**Keywords:** Glucocorticoid, Resistance, Acute lymphoblastic leukemia, Hypoxia, Cell line

## Abstract

**Background:**

Acquired glucocorticoid (GC) resistance remains the main obstacle in acute lymphoblastic leukemia (ALL) therapy. The aim of the present study was to establish a novel GC-resistant B-ALL cell line and investigate its biological characteristics.

**Methods:**

A cell culture technique was used to establish the GC-resistant cell line from the parental cell, NALM-6. Molecular and cellular biological techniques including flow cytometry, MTT assay, western blotting, DNA fingerprinting analysis and whole transcriptome sequencing (WTS) were used to characterize the GC-resistant cell lines. Nude mice were used for xenograft studies.

**Results:**

The GC-resistant cell line, NALM-6/HDR, was established by culturing NALM-6 cells under hypoxia for 5 weeks with a single dexamethasone (Dex) treatment. We subcloned the NALM-6/HDR cell lines, and got 6 monoclone Dex-resistant cell lines, NALM-6/HDR-C1, C3, C4, C5, C6 and C9 with resistance index (RI) ranging from 20,000–50,000. NALM-6/HDR and its monoclone cell line, NALM-6/HDR-C5, exhibited moderate (RI 5–15) to high resistance (RI > 20) to Ara-c; low or no cross-resistance to L-Asp, VCR, DNR, and MTX (RI < 5). STR analysis confirmed that NALM-6/HDR and NALM-6/H were all derived from NALM-6. All these cells derived from NALM-6 showed similar morphology, growth curves, immunophenotype, chromosomal karyotype and tumorigenicity. WTS analysis revealed that the main metabolic differences between NALM-6 or NALM-6/H (GC-sensitive) and NALM-6/HDR (GC-resistant) were lipid and carbohydrates metabolism. Western blotting analysis showed that NALM-6/HDR cells had a low expression of GR and p-GR. Moreover, AMPK, mTORC1, glycolysis and de novo fatty acid synthesis (FAS) pathway were inhibited in NALM-6/HDR when compared with NALM-6.

**Conclusions:**

NALM-6/HDR cell line may represent a subtype of B-ALL cells in patients who acquired GC and Ara-c resistance during the treatment. These patients may get little benefit from the available therapy target of AMPK, mTORC1, glycolysis and FAS pathway.

**Supplementary Information:**

The online version contains supplementary material available at 10.1186/s12935-021-02335-7.

## Background

Although contemporary chemotherapy regimens have improved the long-term survival rate of childhood acute lymphoblastic leukemia (ALL) to nearly 90% [1], the outcome of relapsed/refractory pediatric ALL is still extremely poor. Acquired drug resistance to chemotherapy, especially resistance to glucocorticoids (GCs), remains the main obstacle in ALL therapy [2, 3]. Many efforts were made to seek the mechanism of GC resistance and restore GC sensitivity, but showed little benefit to patients.

Drug-resistant cell lines that are established from drug-sensitive cell lines are the most useful models in tumor research, which have been used to investigate the mechanisms of drug resistance for nearly 50 years [4]. Traditionally, two methods, stepwise dose-escalation continuous exposure and high-concentration pulsatile exposure, are available to acquire drug resistance in cell lines [5]. There is a controversy regarding the traditional drug-exposure method for that the resistant phenotype obtained after a long time (6 ~ 16 months, or more) drug exposure and can only remain for several weeks or months after discontinuation of drug exposure, whether or not they can reflect the clinical setting [4]. Therefore, we constructed a novel and convenient method to establish GC resistant ALL cell lines from GC sensitive ALL cells by mimicking the hypoxic bone marrow (BM) microenvironment [6]. Compared to the traditional methods, the novel method has a high success rate, a single drug exposure, a short duration (5 ~ 6 weeks), and a long maintenance of resistant phenotype without drug re-induction [6].

Hypoxia plays a central role in ALL progression and resistance to therapy [7]. Glycolysis-based metabolic reprogramming enhances multidrug resistance [8]. Previously, we established a T-ALL GC-resistant cell line, CEM-C7/HDR, and reported its biological characteristics [6]. T-ALL and B-ALL are highly heterogeneous disease and may have distinct mechanisms of GC resistance. To clarify the underlying mechanisms and therapeutic targets of hypoxia in relapsed/refractory B-ALL, we established a novel B-ALL GC-resistant cell line, NALM-6/HDR, derived from NALM-6 cell line, and studied the distinct biological characteristics of the cell line.

## Methods

### Cell line and culture conditions

The GC-sensitive B-ALL cell line, NALM-6 was purchased from Shanghai Institute Cell Resources Bank. NALM-6/HDR and NALM-6/H were established from NALM-6 as described previously [6]. All cells were cultured in RPMI 1640 (HyClone, Logan, UT, USA) supplemented with 10% fetal bovine serum (FBS; HyClone) at 37 °C under a humidified atmosphere with 5% carbon dioxide (CO_2_) and 21% oxygen (O_2_; normoxic condition).

### Reagents and antibodies

Dexamethasone (DexA, Propidium iodide (PI), trypan blue, methylcellulose (MethoCult GFH4434) and 3-(4,5-dimethylthiazol-2-yl)-2,5-diphenyltetrazolium bromide (MTT) were obtained from Sigma-Aldrich (St. Louis, MO, US). The Annexin V-PI Kit was purchased from Roche (Mannheim, Germany). Antibodies to ATP-citrate lyase (ACLY), p-ACLY (Ser455), acetyl-CoA carboxylase (ACC), p-ACC (Ser79), fatty acid synthase (FASN), long-chain acyl-CoA synthetase 1 (ACSL1), acetyl-CoA synthetase (AceCS1), AMPK, p-AMPK (Thr172), Glut-1, HKII, LDHA, p-LDHA (Tyr10), 4E-BP1, p-4E-BP1 (Thr37/46), p70S6K, p-p70S6K (Thr389), glucocorticoid receptor (GR), and p-GR (Ser211) were obtained from Cell Signaling Technology (Beverly, MA, USA). Horseradish peroxidase (HRP)–conjugated donkey anti-rabbit antibody and HRP-conjugated sheep anti-mouse antibodies were obtained from Santa Cruz Biotech (Santa Cruz, CA, USA). The β-Actin and GAPDH antibody was purchased from Kangchen Bio-Tech (Shanghai, China).

### Subcloning of ALL cells

As described previously [6], exponentially growing cells were seeded in 6-well sterile plastic culture plates at a density of 5 × 10^2^/ml in methylcellulose RPMI-1640 medium containing 0.9% methylcellulose and 10% FBS at 37 °C under a humidified atmosphere with 5% CO_2_ and 21% O_2_. Monoclonal cells were picked randomly on day 8 of the culture, and cultured in RPMI-1640 complete medium.

### Cell growth and viability assay

Exponentially growing cells were cultured in a 6-well sterile plastic culture plates for seven days. The number of viable cells was counted using heamocytometer and trypan blue staining every day. Doubling time (Td) of cells was calculated according to the following formula: Td (h) = t × lg2/lg(N_t_/N_0_), where t is the time of continuous culture, N_t_ is the final number of cells, and N_0_ is the initial number of cells.

Cell viability was evaluated by MTT assay. Briefly, cells were seeded in 96-well plates. Next, MTT with a final concentration of 0.5 mM was added to the wells and incubated for 4 h at 37 °C. Then acidified SDS solution (10% SDS in 0.01 M HCl) were added to each well and incubated overnight at 37 °C. Finally, the optical density was measured at 570 nm using a multiplate reader (Multiskan Spectrum, Thermo Electron Co., Waltham, MA, USA). Values were obtained by comparing the experimental cells with their respective controls. Mean values were calculated from triplicate cultures.

### Chemosensitivity assays

The chemotherapeutic drugs, Dex, asparaginase (L-Asp), daunorubicin (DNR), vincristine (VCR), arabinoside (Ara-c), and methotrexate (MTX), were purchased from Sigma. All cells were treated with increasing concentrations of different drugs for 48 h, followed by assessment of cell viability by MTT assay. The IC_50_ was calculated by linear interpolation.

### Cell cycle analysis

Cells were harvested 48 h after treatment and fixed overnight in 70% ethanol at 4 °C. The cells were then washed twice with PBS and stained with 5 μg/ml PI in the presence of DNAse-free RNAse (Sigma) for 30 min at room temperature. Then, the cell cycle distribution was analyzed by flow cytometry (Cytomics FC 500 and CXP & Multicycle software, Beckman Coulter Inc., Miami, FL, USA).

### Immunophenotype analysis

Antibodies against the following targets: HLA-DR, cCD3, CD3, CD79α, CD10, CD19, CD20, CD22, CD38, and CD45 (Becton Dickinson Inc., Franklin Lakes, NJ, USA) were used. Positivity for the antigens was determined using a FACSCalibur flow cytometer (Becton Dickinson Inc.).

### DNA fingerprinting analysis

The identity of the NALM-6, NALM-6/HDR and NALM-6/H cell lines was checked using DNA fingerprinting. DNA was prepared from these cells using the Qiagen DNeasy Blood Kit (Qiagen), according to the instructions provided by the manufacturer. The following 22 highly polymorphic short tandem repeat (STR) loci were tested by a multiplex PCR: *Amelogenin*, *CSF1PO*, *D13S317*, *D16S539*, *D5S818*, *D7S820*, *TH01*, *TPOX*, *vWA*, *Penta E*, *Penta D*, *D2S441*, *D2S1338*, *D3S1358*, *D6S1043*, *D8S1179*, *D10S1248*, *D12S391*, *D18S51*, *D19S433*, *D21S11* and *FGA*.

### Whole transcriptome sequencing (WTS) analysis

Total RNA was extracted by Trizol reagent (Invitrogen, Thermo Fisher, Carlsbad, CA, USA) according to the manufacturer's instructions. RNA quality and purity were assessed by RNA 6000 Nano assay on Agilent 2100 Bioanalyzer (Agilent, Santa Clara, CA, USA). RNA-sequencing libraries were acquired on the BGISEQ-500 Platform in total and generated approximately 10.98 Gb per sample. After reads filtering, clean reads were mapped to the reference genome using HISAT. Then, gene expression levels for each sample were calculated with RSEM. Based on the gene expression level, the differentially expression genes (DEGs) between samples can be identified. PossionDis algorithms were used to detect the DEGs. With DEGs, we perform Gene Ontology (GO) classification, Kyoto Encyclopedia of Genes and Genomes (KEGG) pathway classification and functional enrichment.

### Western blotting analysis

Briefly, cells (10^6^) were washed twice in cold PBS and then lysed by RIPA lysis buffer (Bio-Rad). Equivalent amounts of proteins were separated by 8–15% SDS–polyacrylamide gel electrophoresis and transferred to PVDF membranes (0.22 or 0.45 μm, Millipore). Immunoblots were assayed by using primary antibodies. HRP conjugated secondary antibodies were used for detection. The level of the β-Actin protein was used as a control for the amount of protein loaded into each lane.Proteins were visualized by incubation with ECL plus (Millipore). All experiments were conducted independently at least 3 times.

### Animal experiments

Cultured 5 × 10^6^ exponentially growing cells were subcutaneously injected into the right flanks of 6-week-old female BALB/c (nu/nu) nude mice (GemPharmatech Co., Ltd, Nanjing, China), and 0.1 ml of PBS was injected into the left flanks as the control. Tumor size was measured by calipers every 2 days. The approximate tumor volume was calculated using the equation V = (length × width × width)/2. All animals were ear-tagged and monitored individually throughout the experiment. All animal care was in compliance with the guidelines established by the internal Institutional Animal Care and Use Committee and Ethics Committee of Sichuan University. Mice were euthanized by cervical dislocation after anesthetizing with a mixture of 100 mg/kg ketamine and 10 mg/kg xylazine via intraperitoneal injection. Then, the tumor mass was excised, fixed in 10% formalin, and routinely processed for paraffin embedding. Five-millimeter-thick sections were obtained and prepared for standard histopathological examination.

### Statistical analysis

All assays were performed in triplicate, and data are expressed as mean values ± SD. One-way ANOVA was used to compare two groups. A *p*-value < 0.05 was considered to be significant.

## Results

### Establishment of Dex-resistant NALM-6/HDR cell line and its mono-clone cell line

**Fig. 1:**
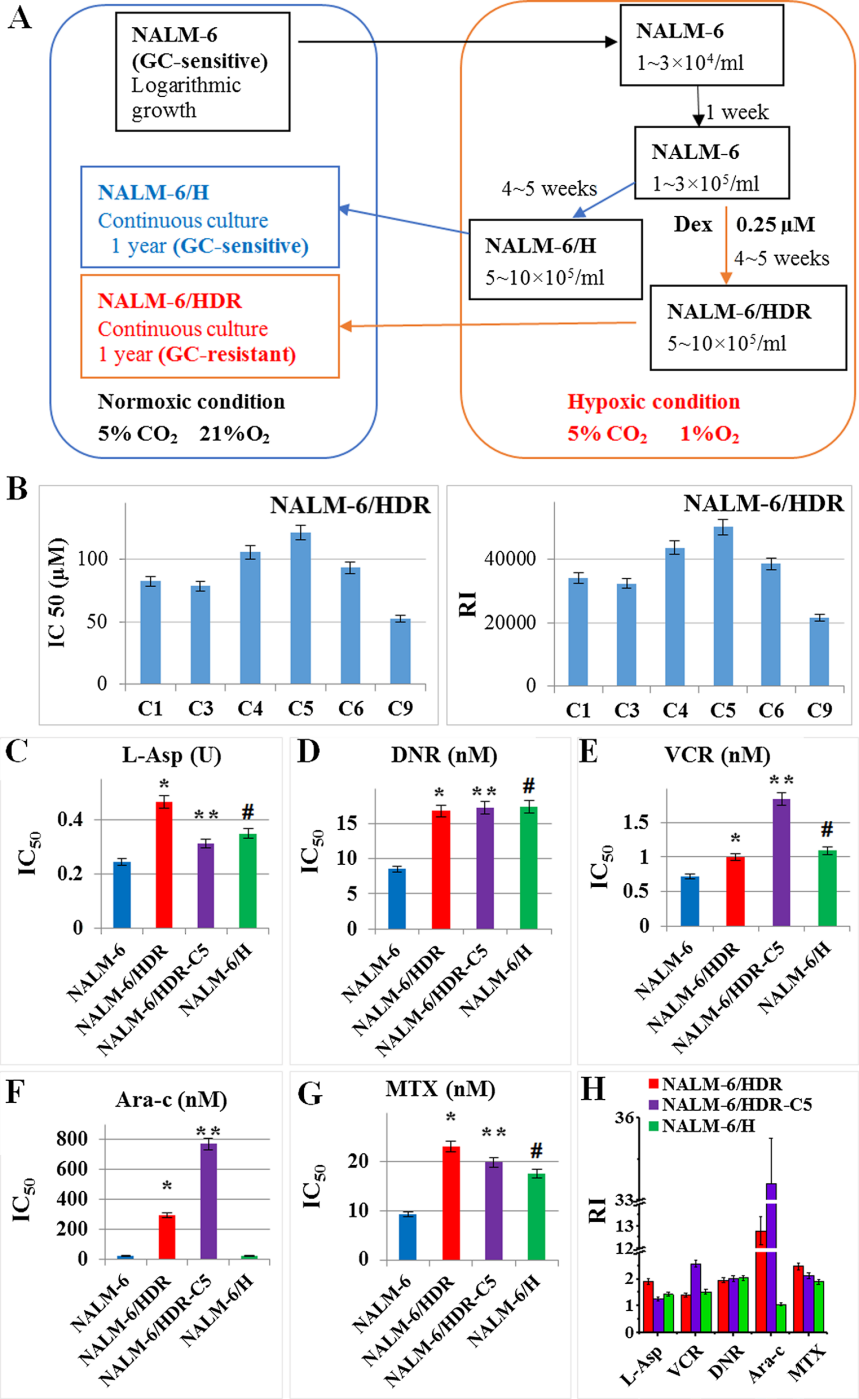
Establishment and resistance characteristics of NALM-6/HDR and its monoclone cell lines. **a** Flow chart for constructing the GC-resistant NALM-6/HDR cell lines. **b** IC_50_ and RI of the subclones of NALM-6/HDR cells to Dex. Cells were cultured with increasing concentrations of Dex for 48 h. Cell viability was evaluated by MTT assays. The IC_50_ values were calculated by linear interpolation. Experiments were performed in triplicate. **c**–**g** IC50 of NALM-6, NALM-6/HDR, NALM-6/HDR-C5, and NALM-6/H cells to L-Asp, DNR, VCR, Ara-c, and MTX. Cells were cultured with increasing concentrations of different drugs for 48 h. Cell viability was evaluated by MTT assays. The IC50 values were calculated by linear interpolation. Experiments were performed in triplicate. **h** RI of NALM-6/HDR, NALM-6/HDR-C5, and NALM-6/H cells to L-Asp, DNR, VCR, Ara-c, and MTX

NALM-6/HDR cell line was constructed following the method we reported previously [6]. Logarithmically growing NALM-6 cells were cultured under hypoxia condition for 5–6 weeks with or without dexamethasone (Dex) treatment. Then, the cells were cultured continuously under normoxic condition and constructed a stable resistant cell line, NALM-6/HDR (Fig. 1a). The IC_50_ and RI of Dex in the NALM-6/HDR cells were 55–70 µM and 25,000–30,000, respectively [6]. All NALM-6/HDR subclones were resistant to Dex with IC_50_ and RI ranging from 50 to 125 µM and 25,000–50,000, NALM-6/HDR-C5 cells were the most resistant cells to Dex among the monoclonal cell lines (Fig. 1b). Unlike the CEM-C7/H [6], the control group, NALM-6/H, which was cultured under the hypoxic condition with no Dex treatment, did not get Dex resistance. The IC_50_ of Dex in the NALM-6, NALM-6/H and its monoclonal cells was 0.002–0.005 µM. GC-resistant cells may get cross-resistance to other drugs [9]. Our results showed that NALM-6/HDR and its monoclonal cell line, NALM-6/HDR-C5, exhibited very high resistance to Dex, moderate (RI 5–15) to high resistance (RI > 20) to Ara-c (Fig. 1f, h); low or no cross-resistance to L-Asp, DNR, VCR, and MTX (RI < 5) (Fig. 1c–h). Although IC_50_ of L-Asp, DNR, VCR and MTX were higher in NALM-6/H than in NALM-6, the RI only ranged from 1.43 to 2.04, which indicated that hypoxia treatment only would not induce drug resistance in NALM-6.

### Morphological and biological characteristics of NALM-6/HDR cells

**Fig. 2:**
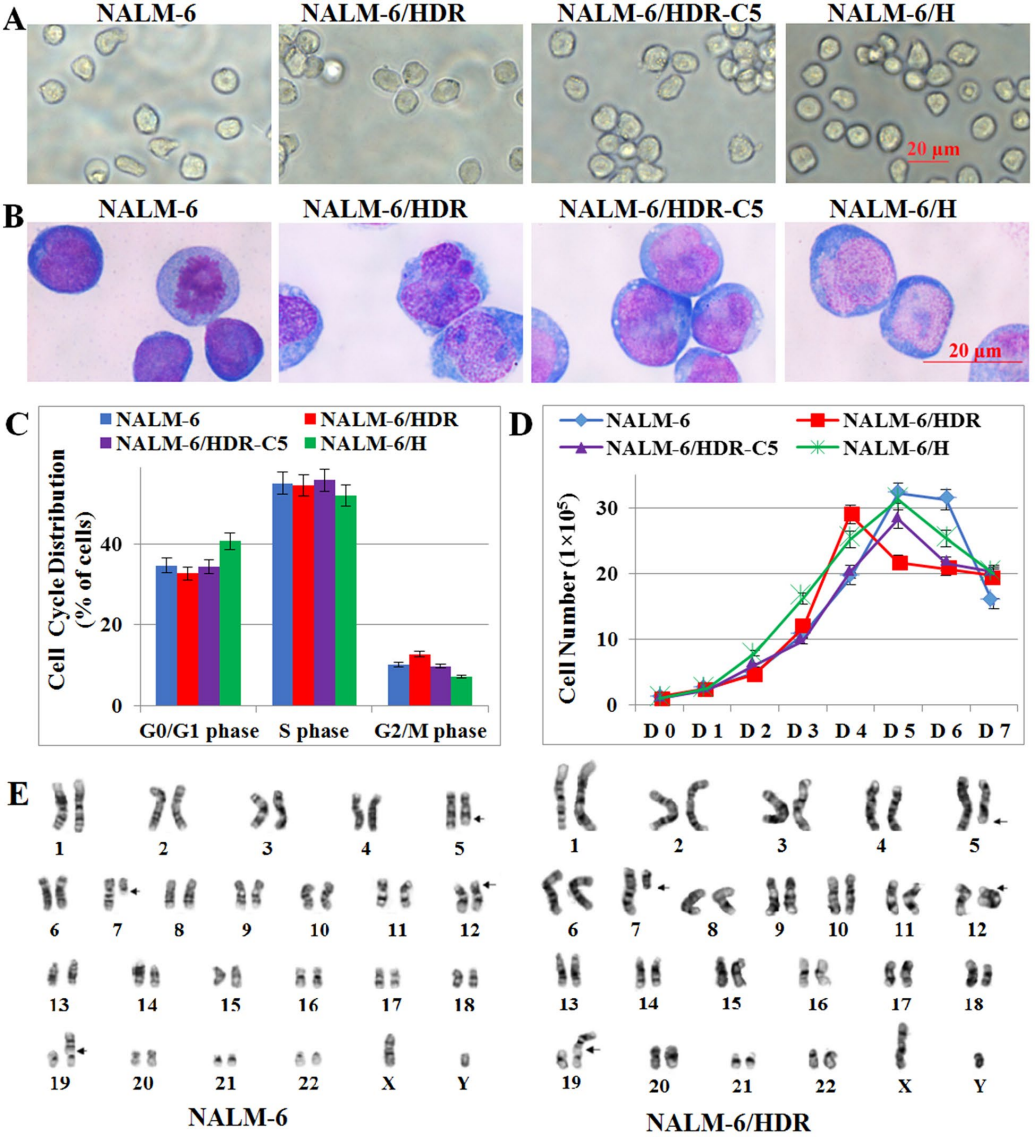
Morphological and biological characteristics of NALM-6/HDR cell line. **a** Morphology of NALM-6/HDR, NALM-6/HDR-C5, NALM-6/H and their parental NALM-6 cells under phase contrast microscopy (× 400 magnification, scale bar 20 µM). **b** Wright-Giemsa staining of cells (× 1000 magnification, scale bar 20 µM). **c** Cell cycle distribution of NALM-6, NALM-6/HDR, NALM-6/HDR-C5, and NALM-6/H cells. Cells were cultured in a 6-well culture plate at 1 × 10^5^/ml, and cell cycle progression was analyzed by PI staining after 48 h. Experiments were performed in triplicate. The percentage of cells in the G_0_/G_1_, S and G_2_/M phase showed no significant difference (*P* > 0.05) compared with each other. **d** Growth curves of NALM-6, NALM-6/HDR, NALM-6/HDR-C5, and NALM-6/H cells. Cells were cultured in a 6-well culture plate at 1 × 10^5^/ml in RPMI-1640 medium with 10% FBS and grown for 7 days. Viable cells were counted using trypan blue staining every day. Experiments were performed in triplicate. **e** G-banding karyotype of NALM-6 and NALM-6/HDR cells. The karyotype according to ISCN (2013) can be described as 46, XY, t(5;12)(q33;p13), t(7;19)(q11.1;p13.3)

The NALM-6/HDR and NALM-6/HDR-C5 cells were grown in suspension as single cells, and Wright-Giemsa staining showed almost no differences in morphology among the cell lines originated from NALM-6 (Fig. 2a, b). All the cells lines showed similar percentages of G_0_/G_1_, S and G_2_/M phase cells, with no statistically significant differences observed (*P* > 0.05) (Fig. 2c). They also exhibited similar growth curves (Fig. 2d). The cell lines all stably proliferated in RPMI-1640 medium containing 10% FBS with population doubling times of 19 ~ 22 h. Chromosomal analysis showed that the karyotype of NALM-6/HDR was 46, XY, t(5;12)(q33;p13), t(7;19)(q11.1;p13.3), the same as NALM-6 (Fig. 2e). NALM-6/HDR and NALM-6 displayed identical immunophenotype with CD10, CD19, CD22, cCD79α and HLA-DR positive (Additional file 1: Fig. S1). STR analysis confirmed that NALM-6/HDR and NALM-6/H were all derived from NALM-6 (Table 1).

**Table 1:** STR analysis of NALM-6, NALM-6/HDR and NALM-6/H

	NALM-6*	NALM-6	NALM-6/HDR	NALM-6/H
Amelogenin	X, Y	X, Y	X, Y	X, Y
CSF1PO	12, 12	12, 13	12, 12	12, 12
D13S317	8, 9, 12	8, 9, 11, 12	8, 12	8, 12
D16S539	9, 10, 11	9, 10, 11	9, 11	9, 11
D5S818	10, 11, 12	11, 12	11, 12	11, 12
D7S820	8, 9, 10	8, 10	8, 10	8, 10
TH01	8, 9	8, 9	8, 9	8, 9
TPOX	8, 10	8, 10	8, 10	8, 10
vWA	15, 16	15, 16	15, 15	15, 15
Penta E	ND	11, 11	11, 11	11, 11
Penta D	ND	8, 14	8, 14	8, 14
D2S441	ND	10, 14	10, 14	10, 14
D3S1358	ND	16, 17	16, 17	16, 17
D6S1043	ND	11, 11	11, 11	11, 11
FGA	ND	22, 23	22, 23	22, 23

*STR profile of NALM-6 was extracted from the database of DSMZ cell bank

### KEGG pathways analysis of DEGs between NALM-6/HDR and NALM-6

**Fig. 3:**
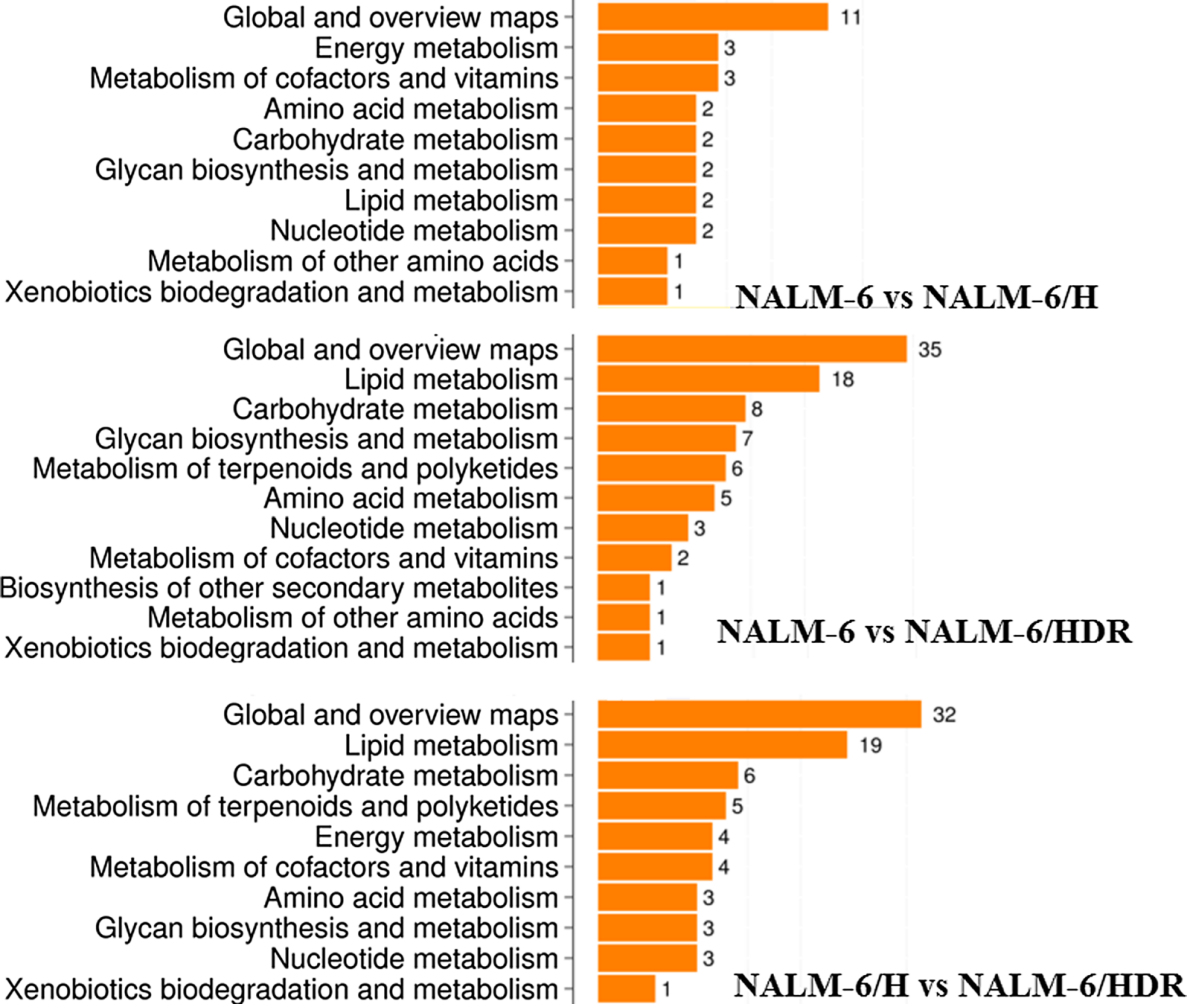
Pathway classifications of DEGs in Metabolism. X axis represents number of DEGs. Y axis represents functional classification in metabolism of KEGG pathway. The main metabolic differences between NALM-6 or NALM-6/H (GC-sensitive) and NALM-6/HDR (GC-resistant) were lipid metabolism and carbohydrates metabolism

To elucidate the underlying mechanisms of GC resistance in NALM-6/HDR, the WTS together with integrated bioinformatics analysis were conducted. Venn diagram was used to display expressed gene between samples, NALM-6, NALM-6/H, and NALM-6/HDR, shown as Additional file 2: Fig. S2a. Summary of DEGs was shown in Additional file 2: Fig. S2b. The results showed a total of 341 DEGs between the NALM-6 and NALM-6/HDR cell lines. Among these, 140 up-regulated and 201 down-regulated DEGs were identified (Additional file 2: Fig. S2b), involving numerous signaling pathways. Metabolic reprogramming is increasingly recognized as a fundamental hallmark of cancer [10]. KEGG pathway classification analysis showed that the main metabolic differences between NALM-6 or NALM-6/H (GC-sensitive) and NALM-6/HDR (GC-resistant) were lipid and carbohydrates metabolism (Fig. 3, Additional file 3: Fig. S3). NALM-6 and NALM-6/H showed a relative similar metabolic character (Fig. 3).

### Expression of proteins associated with fatty acid metabolism

**Fig. 4:**
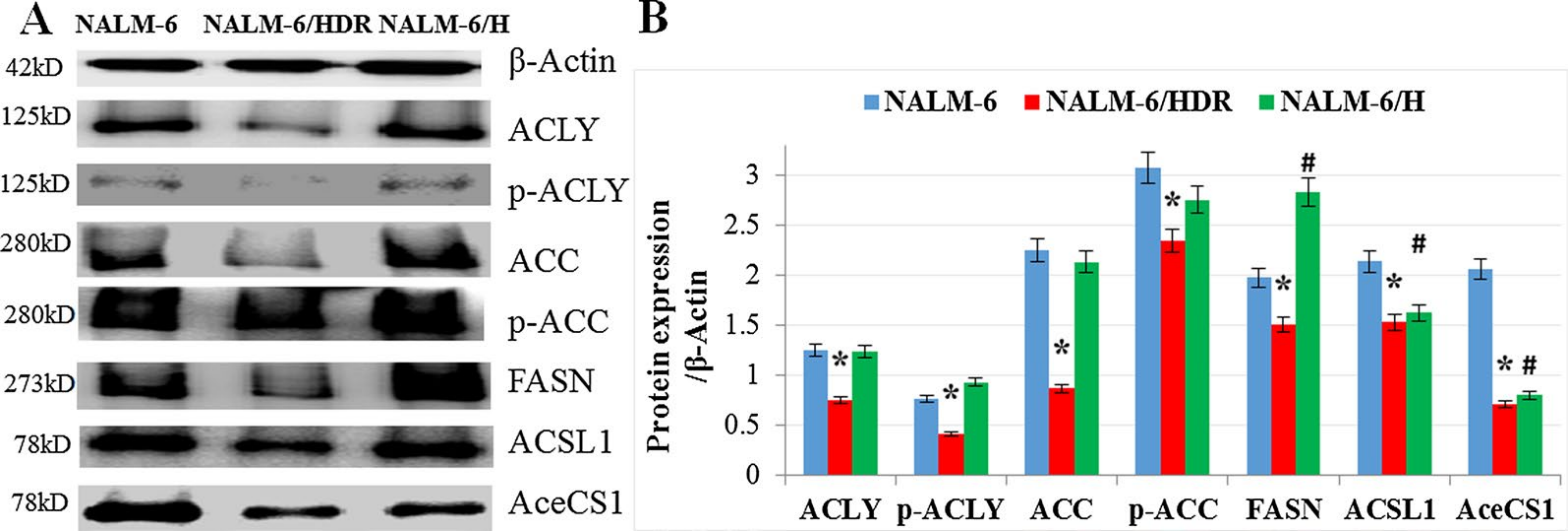
Expression of proteins associated with fatty acid metabolism. **a** Cells were lysed, and extracts were analyzed by western blotting for ACLY, p-ACLY (Ser455), ACC, p-ACC(Ser79), FASN, ACSL1 and AceCS1. β-Actin was used as an internal control. **b** Bar graphs show the ratio of proteins to β-Actin. For all experiments, values of triplicate experiments are shown as the mean ± SD. *: *p* < 0.01 versus NALM-6. ^#^: *p* < 0.01 versus NALM-6

Changes in lipid metabolism, in particular increased de novo fatty acid synthesis (FAS), are recognized as one of the key hallmarks in several cancer cells [11, 12]. Besides, FASN overexpression has shown to be associated with poor prognosis and resistance to chemotherapy [12, 13]. However, in accordance with the results of transcriptome analysis, the results of western blotting showed that the expression of ACLYp-ACLY, ACC, p-ACC, FASN, ACSL1 and AceCS1 were all lower in NALM-6/HDR than in NALM-6 (Fig. 4a, b), which means that NALM-6/HDR cells got a relative low FAS metabolism phenotype accompanying with GC-resistance feature. NALM-6/H showed a relative similar FAS metabolism phenotype with NALM-6, except for a lower expression of ACSL1 and AceCS1 and a higher expression of FASN (Fig. 4a, b). The results hinted that only the GC-resistant NALM-6/HDR cells got a relative low FAS metabolism phenotype.

### Expression of proteins associated with glucose metabolism

**Fig. 5:**
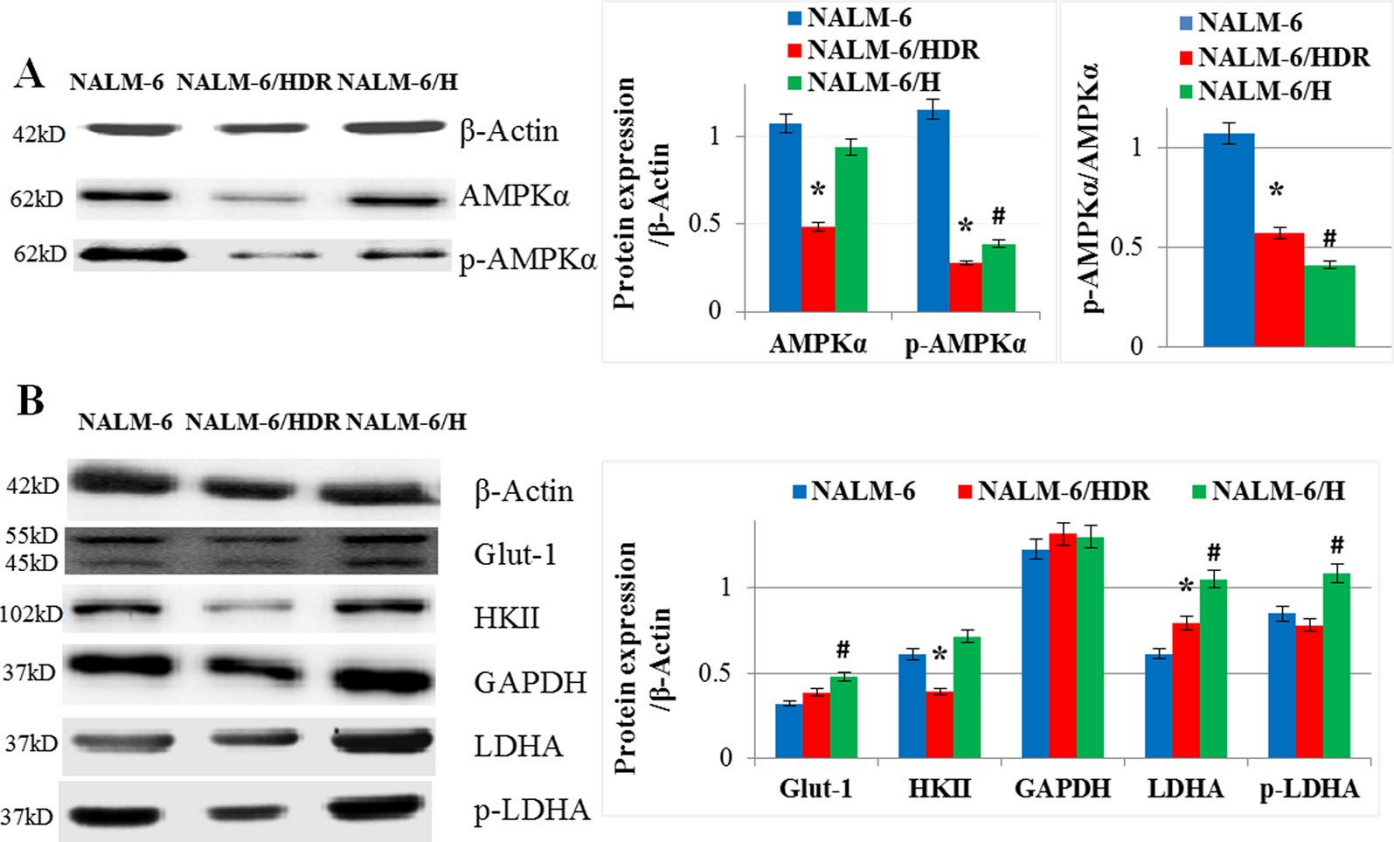
Expression of proteins associated with glucose metabolism. **a** Cells were lysed, and extracts were analyzed by western blotting for AMPK and p-AMPK (Thr172). β-Actin was used as an internal control. Bar graphs show the ratio of proteins to β-Actin and p-AMPK to AMPK. For all experiments, values of triplicate experiments are shown as the mean ± SD. *: *p* < 0.01 versus NALM-6. #: *p* < 0.01 versus NALM-6. **b** Cells extracts were analyzed by western blotting for Glut-1, HKII, GAPDH, LDHA, p-LDHA (Tyr10). β-Actin was used as an internal control. Bar graphs show the ratio of proteins to β-Actin. For all experiments, values of triplicate experiments are shown as the mean ± SD. *: *p* < 0.05 versus NALM-6. ^#^: *p* < 0.05 versus NALM-6

Hypoxia may induce a metabolic switch in energy production, which is characterized by an increase in glycolysis phenotype in cancer cells [14]. We generated NALM-6/HDR cell line from culturing under hypoxia condition for 5 weeks. Therefore, we detected the expression of the energy sensor proteins, AMPK and p-AMPK, and glycolysis-associated proteins. Consistent with the results of CEM-C7/HDR [6], the expression of AMPK and p-AMPK in NALM-6/HDR were both lower than that in NALM-6 (p < 0.01) (Fig. 5a). In NALM-6/H, the expression of p-AMPK was lower than that in NALM-6, and the AMPK expression showed no significant difference (p > 0.05) (Fig. 5a). Moreover, the expression of HKII was reduced (p < 0.01); LDHA were induced(p < 0.01); Glut-1, GAPDH and p-LDHA showed no significant difference (p > 0.05) in NALM-6/HDR when compared with NALM-6 (Fig. 5b). In NALM-6/H, Glut-1, LDHA and p-LDHA were induced (p < 0.01) when compared with NALM-6; HKII and GAPDH showed no difference (p > 0.05) (Fig. 5b); HKII, LDHA and p-LDHA were induced (p < 0.01) when compared with NALM-6/HDR. The results showed NALM-6/HDR got a relative low glycolysis phenotype when compared to NALM-6/H.

### Expression of proteins associated with GC resistance in NALM-6/HDR

**Fig. 6:**
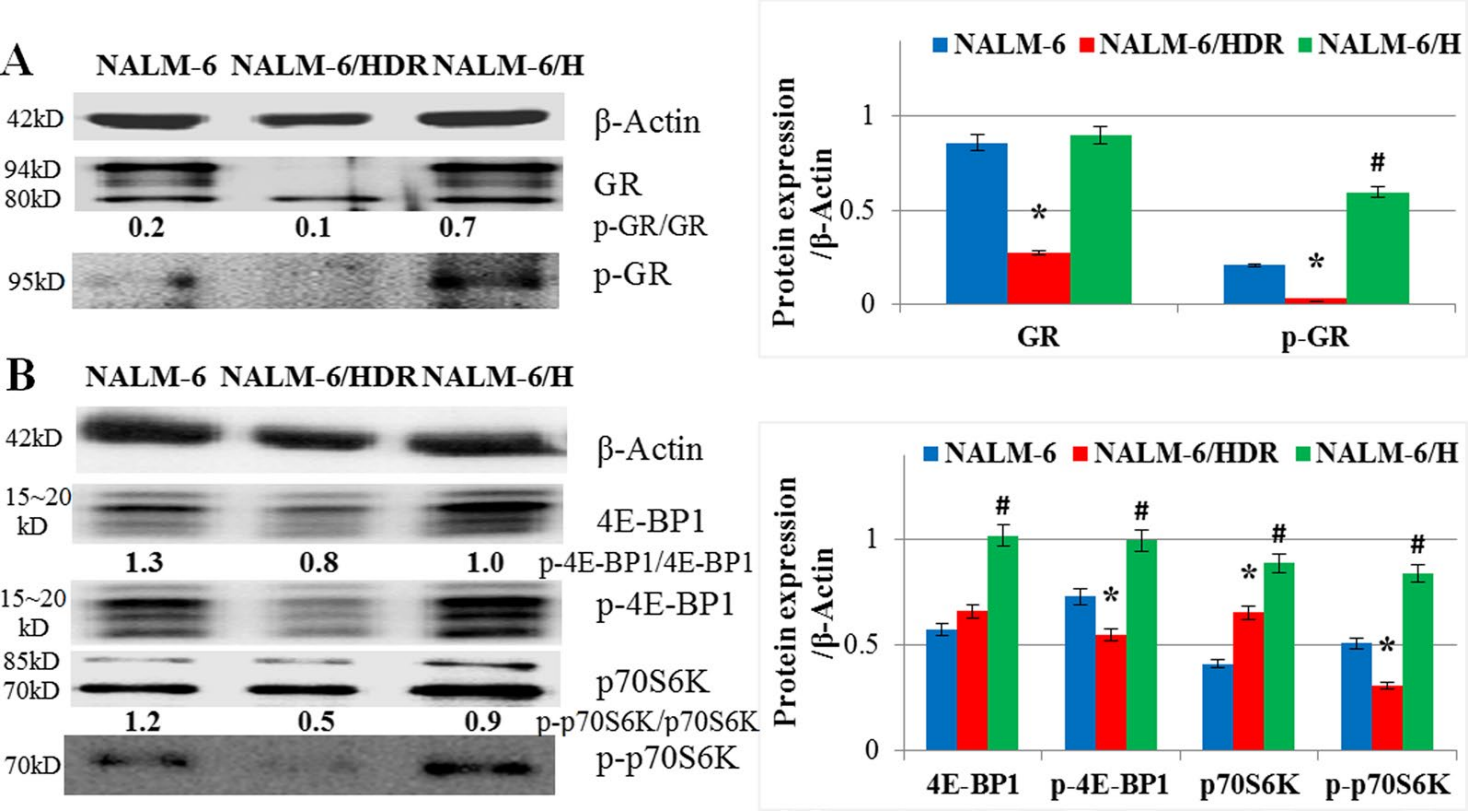
Expression of proteins associated with GC resistance in NALM-6/HDR. **a** Cells extracts were analyzed by western blotting for GR and p-GR (Ser211). β-Actin was used as an internal control. Bar graphs show the ratio of proteins to β-Actin. For all experiments, values of triplicate experiments are shown as the mean ± SD. *: *p* < 0.01 versus NALM-6 and NALM-6/H. #: *p* < 0.01 versus NALM-6 and NALM-6/HDR. **b** Cells extracts were analyzed by western blotting for 4E-BP1, p-4E-BP1 (Thr37/46), p70S6K, and p-p70S6K (Thr389). β-Actin was used as an internal control. Bar graphs show the ratio of proteins to β-Actin. For all experiments, values of triplicate experiments are shown as the mean ± SD. *: *p* < 0.01 versus NALM-6 and NALM-6/H. ^#^: *p* < 0.01 versus NALM-6 and NALM-6/HDR

Loss of GR expression plays a key role in GC resistance and is associated with poor prognosis in leukemia patients [15, 16]. As shown in Fig. 6a, the expression of GR and p-GR (Ser211) were significantly lower in NALM-6/HDR cells than that in NALM-6 and NALM-6/H cells (p < 0.01). However, hypoxia treatment only induced the expression of p-GR and the ratio of p-GR to GR in NALM-6/H (p < 0.01) (Fig. 6a). Dysregulated activation of PI3K-Akt-mTOR pathway may relate to the chemotherapeutic resistance, including GC resistance, in hematological malignances [17, 18]. The expression of the two main downstream effectors of mTOR, 4E-BP1 and p70S6K, and their phosphorylated status p-4E-BP1 (Thr37/46) and p-p70S6K (Thr389), were all induced in NALM-6/H, compared with NALM-6 and NALM-6/HDR cells (p < 0.05) (Fig. 6b). The ratio of p-4E-BP1 (Thr37/46) to 4E-BP1 and p-p70S6K (Thr389) to p70S6K were reduced in NALM-6/H, compared with NALM-6 (Fig. 6b). In NALM-6/HDR, 4E-BP1 showed no significant difference (p > 0.05); p70S6K increased (p < 0.01); p-4E-BP1 (Thr37/46) and p-p70S6K (Thr389) decreased (p < 0.05) when compared with NALM-6. The ratio of p-4E-BP1 (Thr37/46) to 4E-BP1 and p-p70S6K (Thr389) to p70S6K were reduced in NALM-6/HDR (p < 0.05), compared with NALM-6 and NALM-6/H. The results showed that the inhibition of GR and mTOR pathway might be a main character of NALM-6/HDR.

### The tumorigenicity of NALM-6/HDR cells

**Fig. 7:**
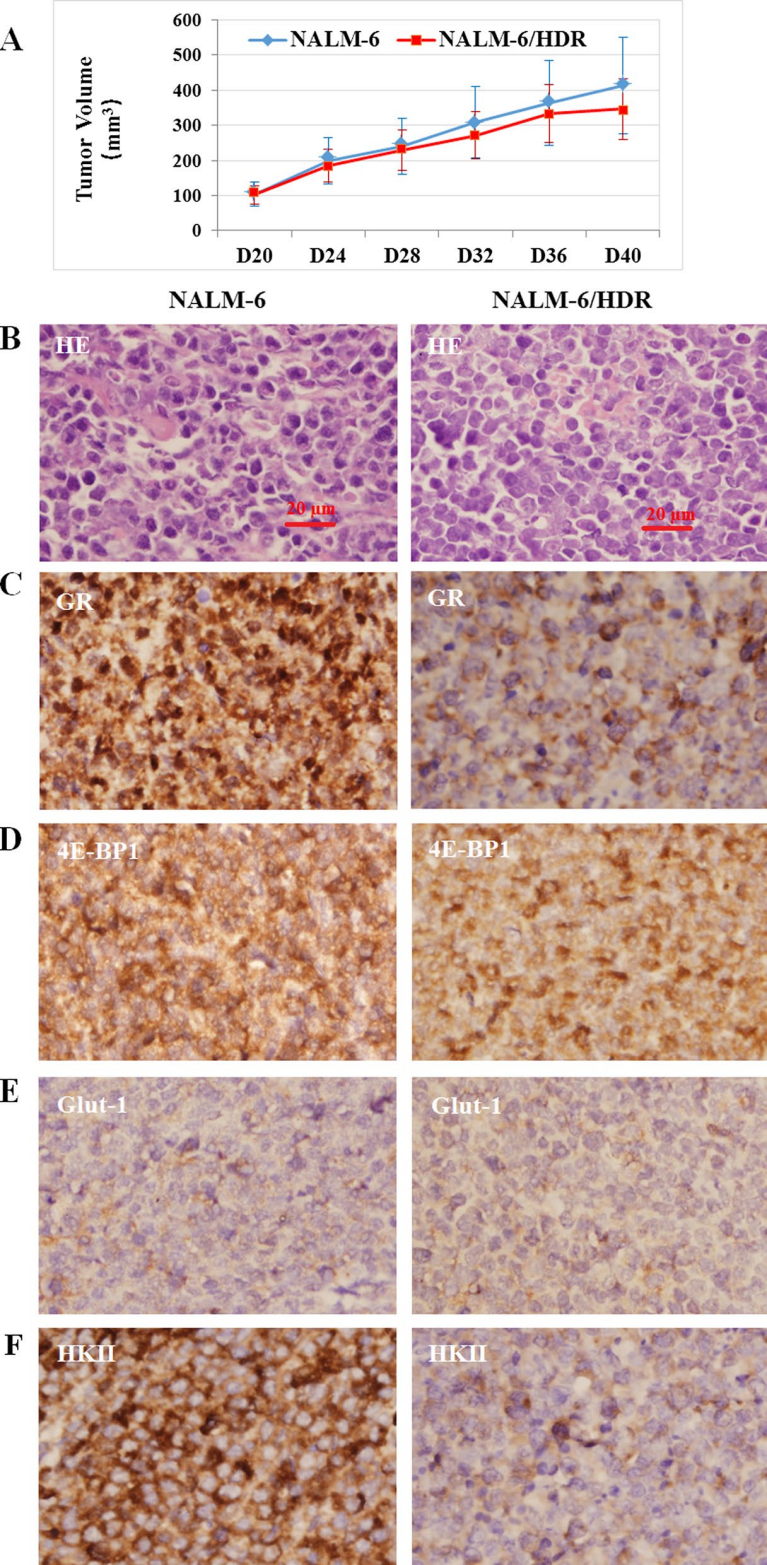
Tumorigenicity of NALM-6/HDR cells. **a** The growth curve of subcutaneous tumors in nude mice. All animal procedures were carried out in accordance with the guidelines established by the internal Institutional Animal Care and Use Committee and Ethics Committee guidelines of Sichuan University. **b** HE staining of the tumors derived from NALM-6 and NALM-6/HDR showed that the xenografts were composed of leukemia cells and blood vessels. Original magnification: × 400 magnification. (c-f) On IHC staining, both xenografts expressed GR, Glut-1, HKII, and 4E-BP1 proteins, with a relative lower expression of GR and HKII in NALM-6/HDR than that in NALM-6

In an in vivo transplantation test, subcutaneous injection of NALM-6 and NALM-6/HDR resulted in the development of tumors in 7 of 12 mice (58%). After 40 days, the mean volume of the tumors generated by subcutaneous injection with NALM-6 and NALM-6/HDR were 413.2 ± 140.0 mm^3^ (n = 4) and 346.9 ± 102.7 mm^3^ (n = 3), respectively (Fig. 7a). Hematoxylin and eosin (HE) staining indicated that the tumor masses were composed of leukemia cells (Fig. 7b). Immunohistochemistry (IHC) staining showed that the expression of GR, Glut-1, HKII, and 4E-BP1 proteins were in consistent with the results of western blotting, the expression of GR and HKII were decreased in NALM-6/HDR obviously (Fig. 7c–f).

## Discussion

Over the last century, studies on patient-derived cancer cell lines have provided crucial insights into the biological processes in tumorigenesis and high-throughput screening for drug development [19]. Molecular alterations that are detected in a drug-resistant cell line, when compared to its drug-sensitive counterpart, can suggest molecular mechanisms underlying drug resistance. Drug resistant cell lines, constructed by the traditional drug-exposure method, have been used in the research on the mechanisms of drug resistance for 50 years, which created a new problem whether or not they can reflect the clinical setting and resole the clinical problems [4]. Furthermore, drugs at a high concentration can kill all parental cells, which make construction of a highly resistant cell line difficult and make construction of a resistant cell line from a highly drug-sensitive cell line more difficult. Therefore, we established a method that matches the manner in which chemotherapy is administered to patients [6]. NALM-6 is highly sensitive to GC, and it is one of the 5 pre-B-ALL cell lines in 100 authenticated leukemia-lymphoma cell lines [20, 21]. Meanwhile, NALM-6 was widely used in the researches on ALL and was recommended as a classic/reference cell line of BCP-ALL [21]. Fortunately, via the novel method, we established the GC-resistant B-ALL cell line, NALM-6/HDR, which is highly resistant to GC with a moderate to high cross-resistant to Ara-C, and shows similar morphology, growth curves, immunophenotype, chromosomal karyotype and tumorigenicity with its parental cell line, NALM-6, and the control cell line, NALM-6/H.

GCs specifically induce apoptosis in malignant lymphoblasts and are thus pivotal in the treatment of lymphoid malignancies, especially in ALL [22]. However, GC resistance remains one of the strongest predictors of relapse in ALL and accounts for most of the treatment failures [2, 3]. Despite decades of clinical practice and laboratory investigation, the exact molecular mechanisms contributing to GC resistance has not been fully uncovered. GCs induce apoptosis by combined with GR and translocated to nucleus and then interaction with GC response elements (GRE), and then activating or suppression transcription of target genes [23, 24]. Initially, most studies showed that low GR protein levels plays a crucial role in GC resistance [15, 16, 23–26], but then the conclusion was rebutted by the results that high GR expression was found also in GC-resistant ALL cells [23, 24, 27, 28]. Anyway, almost all of the GC sensitive ALL cells had a high expression of GR [23]. Recently, loss of GR expression renewed attention on the exploration of GC resistance [15, 16, 23]. Our results showed that NALM-6/HDR expressed a lower GR and p-GR (Ser211) compared to NALM-6 and NALM-6/H in vivo and in vitro. The same, CEM-C7/HDR cells, which were constructed using the same method, showed a low GR expression also [6]. These data implicate reduced GR expression as an important cause of acquired GC resistance.

However, the mechanism of GC resistance is much more complicated than a GR alternation. The level of the GRs expression among ALL patients and derived cell lines appears to be highly heterogeneous [23]. There are several additional mechanisms, triggered by alterations of different signaling pathways, including Notch, Il7R/JAK/STAT, RAS/MEK/ERK, PTEN/PI3K/AKT/mTOR and Ca^2+^ signaling, which cause the metabolic reprogramming, with an enhanced level of glycolysis and oxidative phosphorylation, apoptosis resistance, and multidrug resistance [23]. According to the clinical set, hypoxic stress play a pivotal role in inducing GC resistance in ALL cells [6, 7, 29, 30]. Generally, tumor cells respond to hypoxic stress by upregulating various genes involved in glucose uptake, glycolysis, and angiogenesis, to maintain the cellular metabolic demands; at the same time, translation of “unnecessary” proteins are deregulated to prevent the accumulation of stress-induced unfolded and/or misfolded proteins[29, 31]. Leukemia cells were originated from BM and grown in the hypoxic microenvironment, and then released to the peripheral blood with adequate oxygen. Therefore, leukemic cells, living in both hypoxic and normoxic microenvironments, may have distinct metabolic response to hypoxia from solid tumor cells. A real-time metabolism study on chronic lymphocytic leukemia (CLL) cells showed that circulating CLL cells were primed for hypoxia and showed the same adaptive response upon secondary exposure to hypoxia [32]. Similarly, our results showed that NALM-6/H had a little difference in metabolism when compared to NALM-6. The expression of p-AMPK even decreased in NALM-6/H when compared to NALM-6, which means that there was almost no energy metabolic problem for NALM-6 upon exposure to hypoxia. However, the GC-resistant NALM-6/HDR showed an obvious difference in metabolism, especially in lipid metabolism, when compared to NALM-6 and NALM-6/H.

Cancer cells reprogram lipid metabolism during their malignant progression [12]. Enzymes in de novo FAS pathway, including ACLY, ACC and FASN, are upregulated in numerous cancers [12]. ACLY occupies a privileged position at the crossroads of the metabolism of cancer cells by linking both the glycolysis and lipid metabolism, having been found overexpressed in many aggressive cancers, and might play an important role in cancer relapse [33–35]. The researches on acute myeloid leukemia (AML) showed that low expression of ACLY is associated with favorable prognosis [36, 37]. On the contrary, our results showed that the ACLY and p-ALCY reduced in NALM-6/HDR. ACC is the first rate-limiting enzyme for FAS, which plays a critical role in leukemia progression [38]. ACC was initially considered as an oncoprotein [39], but recent papers showed that ACC might act as a tumor suppressor [38, 40]. ACC1 may protect mice of AML models from leukemia death [38]. Our results also support that ACC might be a tumor suppressor, for the expression of ACC was reduced in NALM-6/HDR obviously with no change in NALM-6/H. FASN, the only human lipogenic enzyme available for de novo FAS, has been widely reported to promote cancer progression [41, 42]. Upon hypoxic stress, different cancer cells had different cell-type-specific regulation of FASN, increased, decreased or unaffected [42]. A recent paper reported that FASN was upregulated in relapsed ALL patients; moreover, FASN inhibition may help to overcome glucocorticoid resistance [43]. FASN inhibition may sensitize AML cells to differentiation therapy [13]. On the contrary, our results showed that hypoxic stress induced FASN expression in NALM-6/H; however, FASN was reduced in GC resistant NALM-6/HDR cells. To be used in metabolic pathways, fatty acid must be activated by ACSLs, which convert free fatty acid to acyl-CoA [12]. ACSL1 may play a potential oncogenic role in colorectal and breast cancer and play a potential tumor suppressor in lung cancer [44]. AceCS1 catalyzes the conversion of acetate to CoA to acetyl-CoA and promote the FAS. Cancer cells display an up-regulated capability to utilize acetate [45]. Our results showed that ACSL1 and AceCS1were reduced in both NALM-6/HDR and NALM-6/H, which might be a stress reaction to hypoxia. Therefore, the results on GC resistant NALM-6/HDR and GC sensitive NALM-6/H may raise some new points about the roles of de novo FAS in relapsed/refractory leukemia. The relative low expression of ACLY, ACC, and FASN in GC-resistant NALM-6/HDR cells pointed out that targeting FAS might not a good idea for certain patients of relapsed/refractory leukemia.

Reprogramming of carbohydrate metabolism, particularly increased glycolysis, has been recognized as a hallmark of cancer [46] and plays a key role in cancer progression in different cancers [8, 10, 47], which forms the basis of tumor imaging by positron emission tomography. Targeting glycolytic metabolism has become a potent therapeutic strategy to suppress cancer progression [14, 48]. Hypoxic stress may influence the energy metabolism and then induce glycolysis, which may contribute to chemoresistance [14, 31, 49]. Our results showed that hypoxic stress did not induce the expression of AMPK and p-AMPK. On the contrary, the expression ratio of p-AMPK and AMPK reduced in NALM-6/HDR and NALM-6/H. AMPK plays a paradoxical dual role in ALL survival and progression [49–51]. Here, decreased expression of AMPK and p-AMPK might contribute to GC resistance in NALM-6/HDR. NALM-6/HDR showed a decreased HKII and an increased LDHA in vitro* or *in vivo. NALM-6/H showed an increased Glut-1, LDHA and p-LDHA. These results suggest that AMPK and glycolysis pathway may not be a therapeutic target in GC resistant NALM-6/HDR. A recent paper reported that cancer cells preferred to uptake glutamine while myeloid cells in tumor microenvironment (TME) took up the most glucose [52]. Therefore, targeting glycolysis may enhance or impair tumor-related inflammation by disrupting the metabolic reprogramming of immune cells in TME, but not cancer cells [52]. A program of mTORC1-driven uptake of glutamine may suppress the expression of glycolysis-related genes and glucose metabolism in cancer cells [52]. We observed mTOR pathway was inhibited in NALM-6/HDR cells. Chronic and prolonged hypoxic exposure results in repression of mTORC1 signaling [53]. Initially, most researchers reported that mTOR signaling activation may contribute to chemoresistance and ALL progression [17, 23, 54]. On the contrary, a recent paper showed that activation of mTORC1 pathway may help suppress the drug resistance of T-ALL in hypoxic niches [55]. It is intriguing that while AMPK functions as an antagonist of mTOR [56]; both AMPK and mTOR signaling pathways were inhibited in NALM-6/HDR cells, which is consistent with the findings in CEM-C7/HDR [6]. The orchestration of the cellular metabolism is not conducted by a single leader; the complex interplay between AMPK, mTOR, and other signaling network help to meet the conflicting self-requirement cancer cells [56]. Therefore, the relative low expression of AMPK and mTOR pathway is a distinct feature of NALM-6/HDR, which might be another key point to understand the mechanisms of GC resistance in ALL [6], and NALM-6/HDR may provide a novel model that represents a subtype of GC-resistant B-ALL cells.

## Conclusions

Here, we constructed a new GC-resistant B-ALL cell line by mimicking the clinical setting in vivo. Compared with its parental cell line, NALM-6, the novel cell line, NALM-6/HDR, has a low expression of GR, a common feature of GC-resistant ALL cells. Moreover, NALM-6/HDR has some distinct characters, including depression in AMPK, mTORC1, glycolysis and FAS pathway, which may hint that the previous therapeutic strategies, such as targeting AMPK, mTORC1, glycolysis and FAS pathway, could not reverse the GC resistant, or even induce some subtype of ALL aggravation. In addition, NALM-6/HDR got cross-resistance to Ara-C. Therefore, NALM-6/HDR may represent a novel cell type in B-ALL patients who acquired GC and Ara-c resistance during the treatment. NALM-6/HDR may serve as valuable in vitro and in vivo tools for further investigation on the potential mechanisms and therapeutic targets of relapsed/refractory B-ALL, especially the roles of the hypoxic TME in GC resistance.

## Supplementary Information

**Additional file 1: Fig. S1.** Immunophenotypic characteristics of NALM-6/HDR cell line. The immunophenotype was analyzed using a FACSCalibur flow cytometer. NALM-6/HDR and NALM-6 displayed identical immunophenotype with CD10, CD19, CD22, cCD79α and HLA-DR positive.

**Additional file 2: Fig. S2. **Venn diagram and DEGs analysis on NALM-6, NALM-6/H, and NALM-6/HDR. **a** Venn diagram was used to display expressed gene between samples. **b** Based on the gene expression level, PossionDIS algorithms was used to detect the DEGs. X axis represented the sample. Y axis represented the DEGs.

**Additional file 3: Fig. S3. **Heatmap of DEGs and the DEGs in metabolism pathway between NALM-6 and NALM-6/HDR cells. **a** X axis represented the sample, NALM-6 and NALM-6/HDR. Y axis represented the DEGs. The color represents the log10 transformed gene expression level. The dark color means the high expression level while the light color means the low expression level. **b** The metabolic functional enrichment results of NALM-6 compared with NALM-6/HDR after hierarchical clustering were shown in the table.

## Data Availability

The datasets used and analyzed during the current study are available from the corresponding author on a reasonable request.

## Abbreviations

ALL: Acute lymphoblastic leukemia
ACC: Acetyl-CoA carboxylase
ACLY: ATP-citrate lyase
AceCS1: Acetyl-CoA synthetase
ACSL1: Long-chain acyl-CoA synthetase 1
AML: Acute myeloid leukemia
BM: Bone marrow
Dex: Dexamethasone
FAS: Fatty acid synthesis
FASN: Fatty acid synthase
GCs: Glucocorticoids
GR: Glucocorticoid receptor
PDL: Population doubling level
STR: Short tandem repeat
Td: Doubling time
TME: Tumor microenvironment
