# Supplementary figures and images for: Characterization of a novel glucocorticoid-resistant human B-cell acute lymphoblastic leukemia cell line, with AMPK, mTOR and fatty acid synthesis pathway inhibition
Source: Cancer Cell Int. 2021 Nov 25;21:623. doi: 10.1186/s12935-021-02335-7 (PMC8614043; doi:10.1186/s12935-021-02335-7)

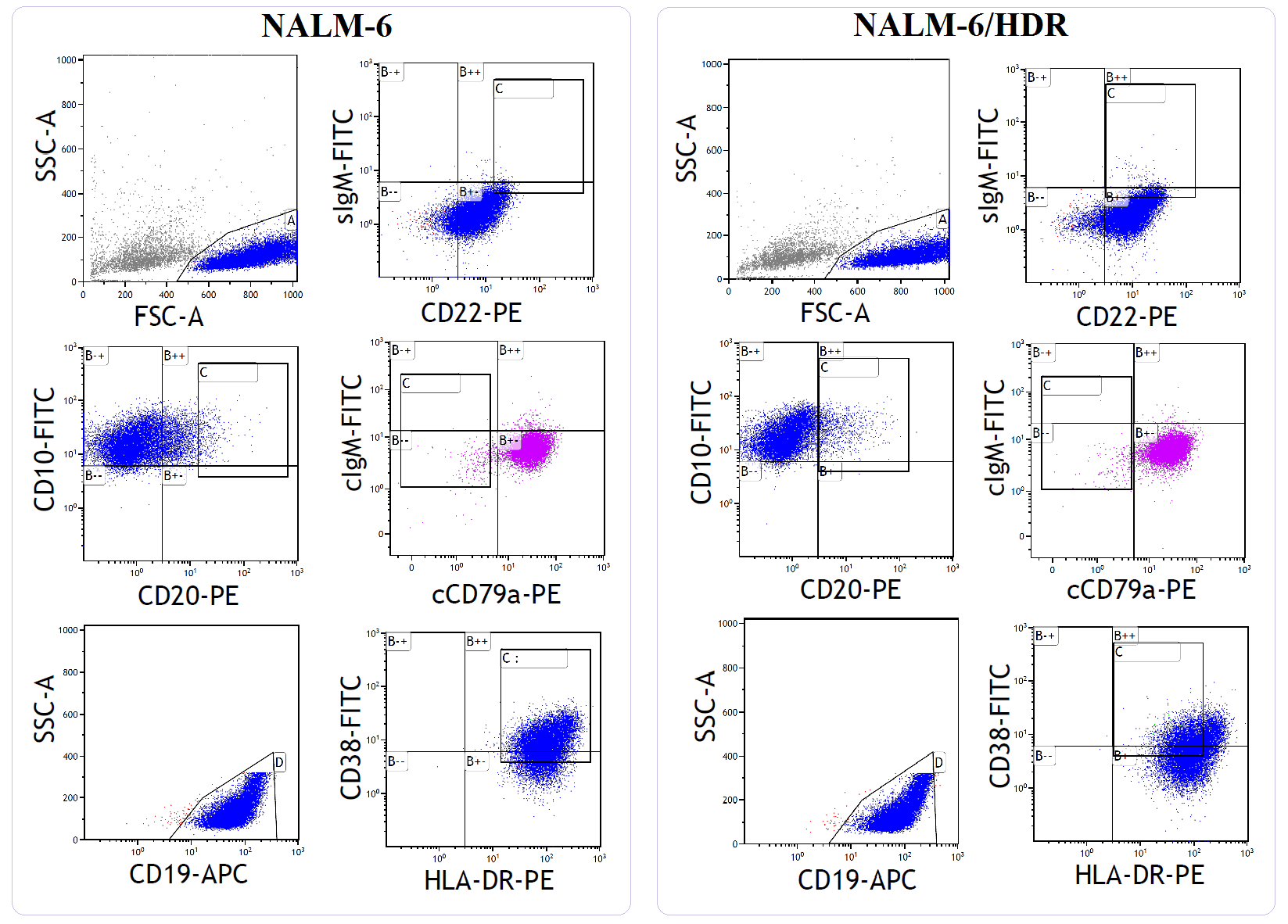

Supplement: Supplementary file 1 — Additional file 1: Fig. S1. Immunophenotypic characteristics of NALM-6/HDR cell line. The immunophenotype was analyzed using a FACSCalibur flow cytometer. NALM-6/HDR and NALM-6 displayed identical immunophenotype with CD10, CD19, CD22, cCD79α and HLA-DR positive. [file 12935_2021_2335_MOESM1_ESM.tif]

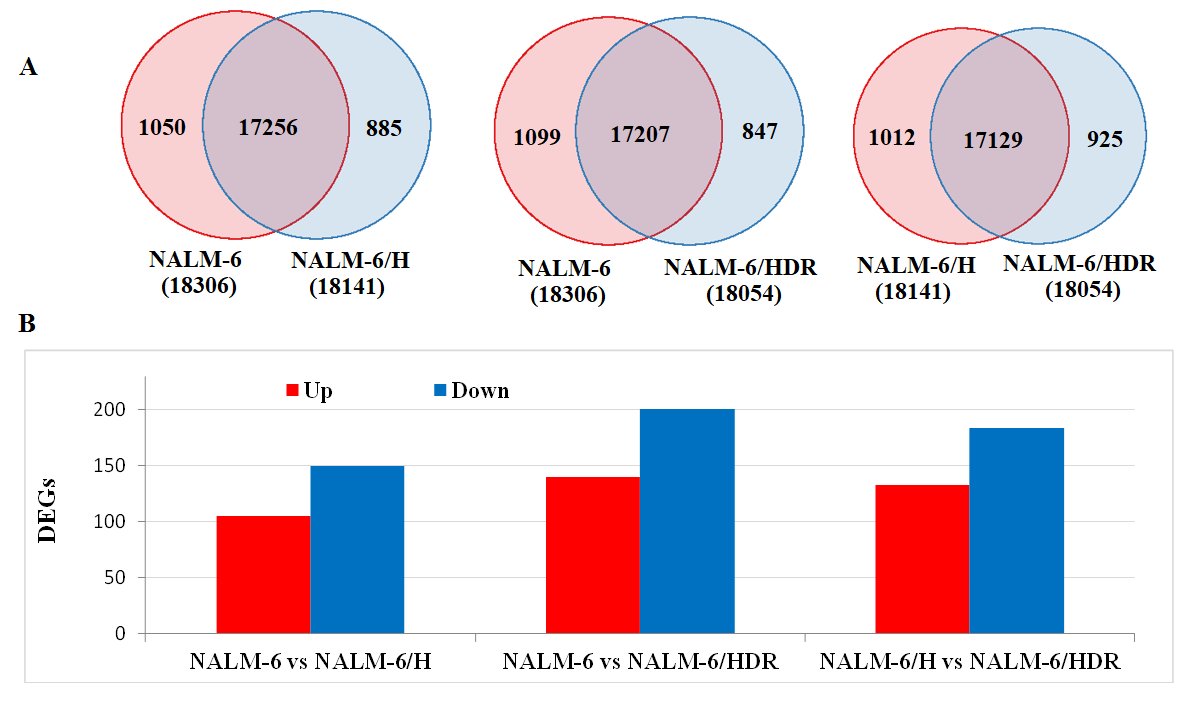

Supplement: Supplementary file 2 — Additional file 2: Fig. S2. Venn diagram and DEGs analysis on NALM-6, NALM-6/H, and NALM-6/HDR. a Venn diagram was used to display expressed gene between samples. b Based on the gene expression level, PossionDIS algorithms was used to detect the DEGs. X axis represented the sample. Y axis represented the DEGs. [file 12935_2021_2335_MOESM2_ESM.tif]

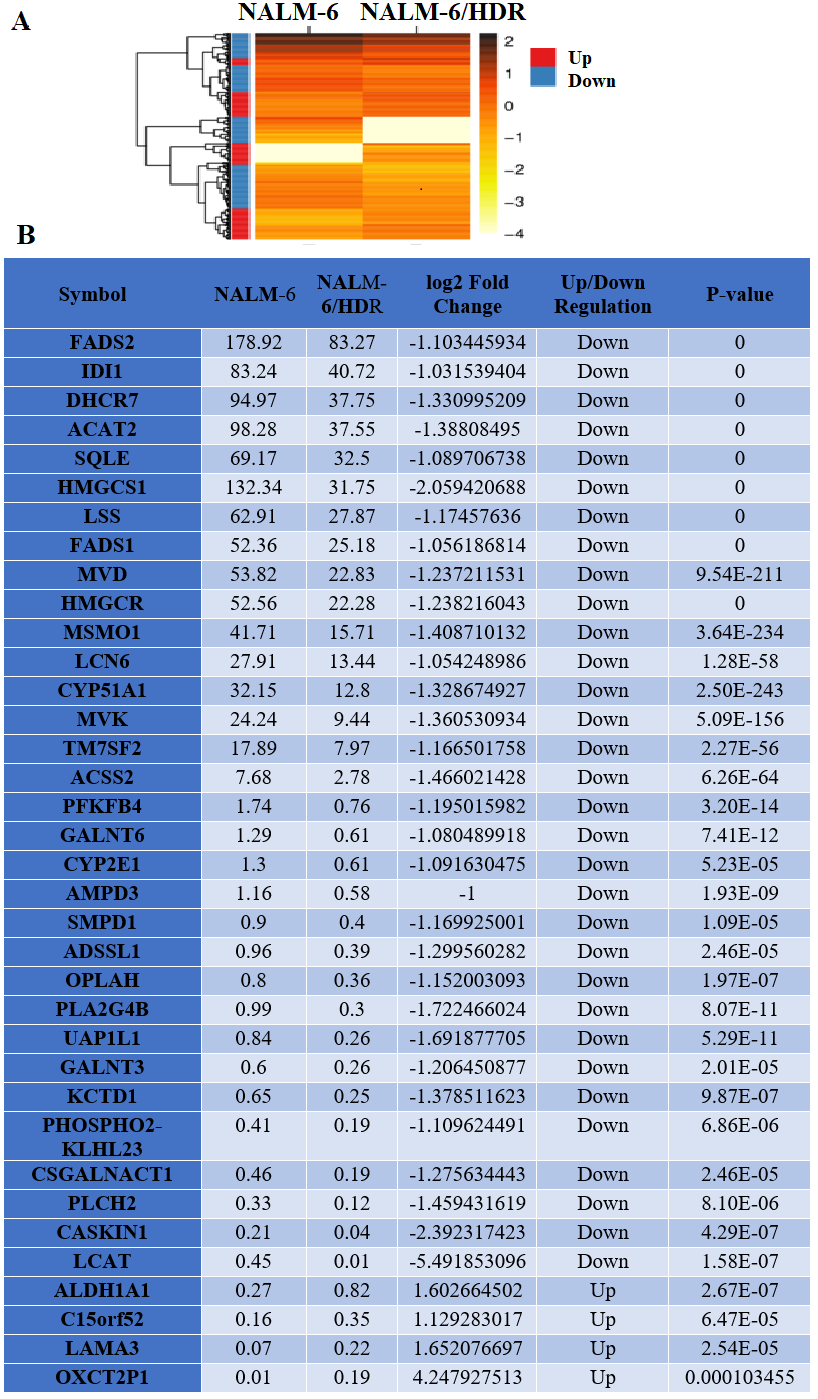

Supplement: Supplementary file 3 — Additional file 3: Fig. S3. Heatmap of DEGs and the DEGs in metabolism pathway between NALM-6 and NALM-6/HDR cells. a X axis represented the sample, NALM-6 and NALM-6/HDR. Y axis represented the DEGs. The color represents the log10 transformed gene expression level. The dark color means the high expression level while the light color means the low expression level. b The metabolic functional enrichment results of NALM-6 compared with NALM-6/HDR after hierarchical clustering were shown in the table. [file 12935_2021_2335_MOESM3_ESM.tif]
